# Broadband tunable integrated CMOS pulser with 80-ps minimum pulse width for gain-switched semiconductor lasers

**DOI:** 10.1038/s41598-017-07138-3

**Published:** 2017-07-31

**Authors:** Shaoqiang Chen, Shengxi Diao, Pengtao Li, Takahiro Nakamura, Masahiro Yoshita, Guoen Weng, Xiaobo Hu, Yanling Shi, Yiqing Liu, Hidefumi Akiyama

**Affiliations:** 1 School of Information Science and Technology, East China Normal University, 500 Dongchuan Road, Shanghai, 200241 China; 2 Institute for Solid State Physics, The University of Tokyo, 5-1-5 Kashiwanoha, Kashiwa, Chiba 277-8581 Japan; 3 Research Center for Photovoltaics, National Institute of Advanced Industrial Science and Technology, 1-1-1 Umezono, Tsukuba, Ibaraki, 305-8560 Japan

## Abstract

High power pulsed lasers with tunable pulse widths are highly favored in many applications. When combined with power amplification, gain-switched semiconductor lasers driven by broadband tunable electric pulsers can meet such requirements. For this reason, we designed and produced a low-cost integrated CMOS pulse generator with a minimum pulse width of 80 ps and a wide tuning range of up to 270 ns using a 40-nm microelectronic process technique. We used this pulser to drive a 1.3-µm semiconductor laser diode directly, and thereafter investigated the gain-switching properties of the laser system. The optical pulses consist of a spike followed by a steady state region. Tuning the width of the electrical pulse down to approximately 1.5 ns produces optical pulses consisting only of the spike, which has a minimum pulse-width of 100 ps. Moreover, the duration of the steady state can be tuned continuously by tuning the electrical pulse width, with a peak power of approximately 5 mW. The output voltage of the electric pulser has a tuning range of 0.8–1.5 V that can be used to directly drive semiconductor laser diodes with wavelengths in the near-infrared spectrum, which are suitable for power amplification with rare-earth doped fiber amplifiers.

## Introduction

High power pulsed lasers, including the currently used commercial femtosecond mode-locked Ti:sapphire lasers, microsecond and nanosecond Q-switched lasers, and picosecond and femtosecond fiber lasers, have been deployed in many applications in areas such as pulse laser deposition (PLD)^1–3^, laser processing^4–7^, and laser ablation^8–10^. Different applications typically require pulsed lasers with different pulse energies and durations. For example, in the case of laser machining, different hole sizes and shapes on the surface of a material corresponding to laser ablation with different pulse widths have been reported^10^. In the case of PLD, it has also been reported that the crystal quality and surface morphology of the deposited thin films vary for different pulse widths^1–3^. Consequently, in order to ensure flexible and precise processing, high power pulse-width-tunable lasers are essential. The commercially used Ti:sapphire and fiber based high power pulsed lasers typically have fixed pulse durations and are not pulse-width-tunable, which in some cases limits their applications.

In high-power fiber lasers, high output powers are achieved through power amplification of seed lasers with rare-earth-doped-fiber amplifiers; therefore, one of the approaches towards the realization of high-power pulse-width-tunable lasers is to manufacture a pulse-width-tunable seed laser. Gain-switched semiconductor lasers are good candidates for use as seed lasers^11–17^, owing to their many unique properties including their simplicity, frequency-tunable capability, and their low-cost and subsequent mass-production potential. Several studies on the fabrication of pulsed fiber lasers using gain-switched semiconductor lasers have therefore been carried out^18–20^.

In gain-switched semiconductor laser systems, the structural parameters of the diode have significant effects on the laser’s gain-switching characteristics. Short optical pulses can thus be generated by improving the structural and material properties of the laser diodes^21–24^. Since the laser diodes are directly modulated by the electric pulsers, the properties of the electrical output pulse are also important in determining the pulse lasing performance of the laser system. As a result, the development of practical, low-cost, compact, pulse-width-tunable electrical pulse generators is also crucial. Moreover, even with long electrical pulse excitations, short gain-switched pulses can be obtained by applying several post treatment methods, such as compression and spectral filtering^13–17^, to the output pulse shape. However, the simplest way of obtaining gain-switched pulses is direct generation of optical pulses from a semiconductor laser. For this reason, short electric pulse generators based on low-cost semiconductor transistors and integrated circuits are in demand.

Generating short optical pulses from semiconductor laser diodes typically requires electrical pulses tens of picoseconds in duration. If the pulse width is tunable, the frequency range covered will be very wide. Although GaAs or SiGe processes^25, 26^ and solid-matching can be utilized to realize the required pulses, both processes are expensive and not easily integrated with the whole system when compared with complementary metal-oxide-semiconductor (CMOS) technology^27, 28^. In spite of the low cutoff frequency of standard CMOS technology and the resulting difficulty in generating very narrow pulses^28–30^, with improvements to the CMOS process, the cutoff frequency is increased up to a point where it almost meets the requirements. Moreover, through circuit design and current-driven techniques, the laser driving capability can be improved.

In this paper, we designed and constructed a low-cost CMOS based electric pulser with tunable pulse widths within the range of 0.08–270 ns, and tunable output voltages within the range of 0.8–1.5 V. We used this pulser to directly drive a semiconductor laser diode and investigated the gain-switching properties, thereby demonstrating the pulse-width-tunable characteristics of the total laser system. The output pulse shape depends on the electrical pulse, with a tunable duration within the range of 0.1–270 ns. CMOS integrated circuits have therefore been verified as a good choice for use in low-cost and compact electric pulsers for gain-switched semiconductor laser diodes.

## Materials and Methods

**Figure 1:**
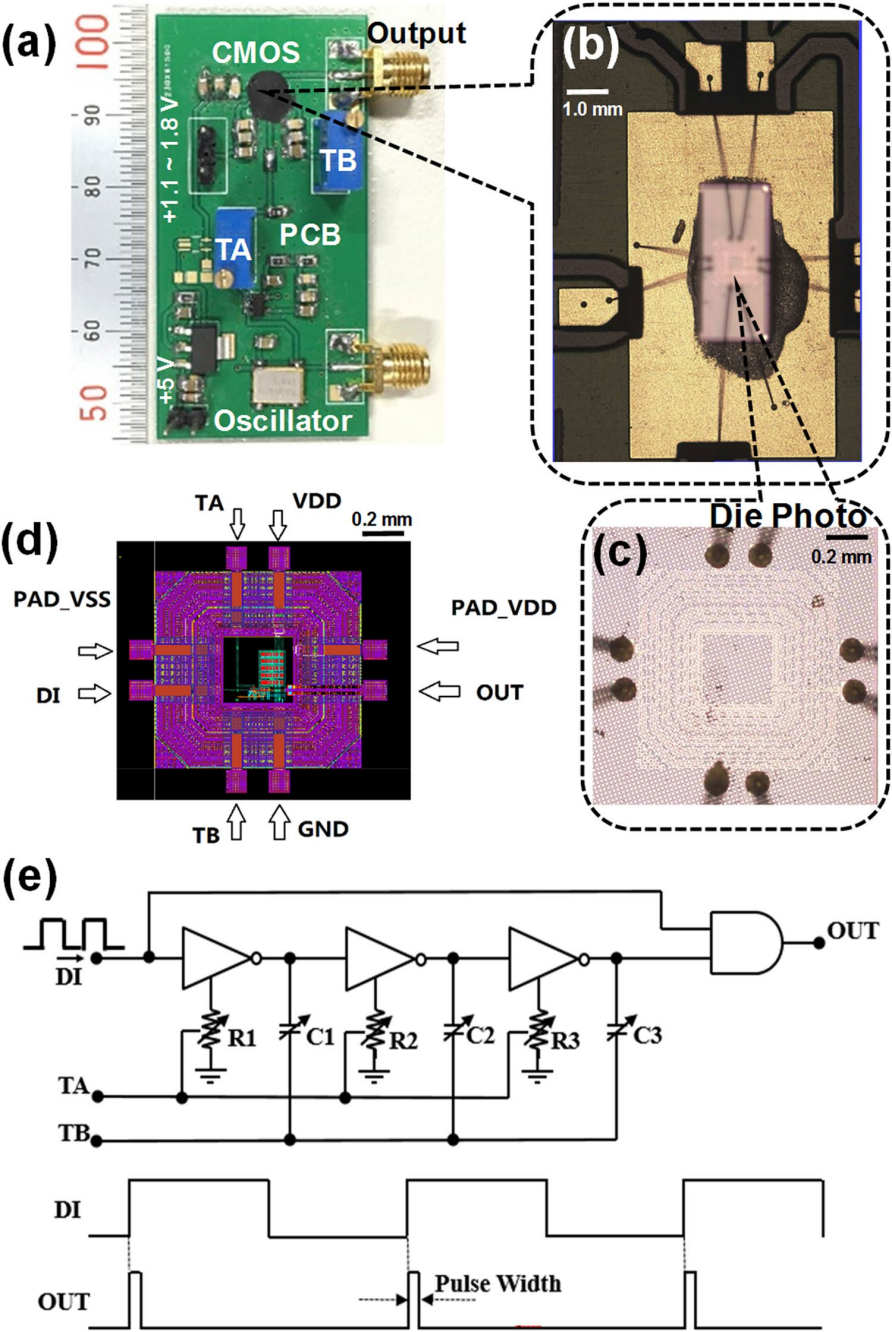
Schematic design and images of the electric pulser. (**a**) Image of the lab-made electric pulser on a printed circuit board (PCB). (**b**) Image of the CMOS pulse generator taken before packaging on the PCB. (**c**) Enlarged image of the CMOS pulse generator. (**d**) Design layout of the CMOS pulse generator. (**e**) Schematic design of the CMOS circuit. TA: Tuning A, TB: Tuning B, DI: Data Input, VDD: Voltage Drain Drain, VSS: Voltage Source Source, GND: Ground.

First, we designed and produced a low-cost CMOS pulse generator using a 40 nm process technique, in a standard IC foundry (Semiconductor Manufacturing International Corporation). We then designed the peripheral circuit and converted the CMOS pulse generator into a pulse-width-tunable electric pulser packaged on a printed circuit board (PCB). Figure 1(a) shows an image of the electric pulser captured using a digital camera. A nanosecond oscillator with an operating frequency of 1.8 MHz was used to trigger the CMOS pulse generator. Figure 1(b) shows an image of the CMOS pulse generator, captured before packaging on the PCB. Figure 1(c) shows an enlarged image of the CMOS pulse generator, Fig. 1(d) shows its design layout, and Fig. 1(e) shows the schematic design of the CMOS circuit. The CMOS pulse generator consists of three cascaded, current-tunable inverters, and a tunable metal-oxide-semiconductor capacitor (MOSCAP). This tunable current represents a virtual resistor, *R*, and the tunable MOSCAP represents a tunable capacitance, *C*. Consequently, a tunable delay of τ = *RC* can be easily achieved using both tuning mechanisms. A pulse width equal to τ can be generated by performing a logical AND operation on the delayed square wave and the original signal. A detailed schematic of the tunable pulse generator is given in the Supplementary Information.

As laser diodes generally have an input impedance of around 50 Ω, a driver is needed to achieve a high voltage amplitude and narrow pulse width. Using calculations and simulations, aspect ratios of 100 µm/40 nm for PMOS transistors and 40 µm/40 nm for NMOS transistors were selected to form the inverter used to drive the external 50 Ω laser diode. The die photo (Fig. 1(c)) of the CMOS pulse generator shows that the whole pulse generator, including the PAD connectors, which only occupy an area of 1 mm^2^. The pulser is driven with a 5 V DC current source, and the integrated CMOS pulse generator is driven with DC voltages between 0.8 and 1.5 V. The output pulse shapes of the electric pulser were measured using a high-speed oscilloscope.

We used this pulser to directly drive a commercial semiconductor distributed feedback (DFB) laser diode in order to investigate the gain-switching properties and demonstrate the pulse-width-tunable characteristics of the total laser system. The laser diode is in a pigtail package, with a lasing wavelength of 1.3 µm. We directly applied the electrical output from the pulser onto the semiconductor laser diode. The waveforms of the output pulse were measured using a high-speed photodetector combined with a high-speed oscilloscope, and the laser powers were measured using a power meter.

The oscilloscope used for this study is an Agilent 86100D with an electrical module, Agilent 86117 A, with a bandwidth of 50 GHz, rise time of 7 ps, and jitter of less than 1 ps. The photodetector is a New Focus Model 1014 with a bandwidth of 45 GHz.

## Results and Discussion

**Figure 2:**
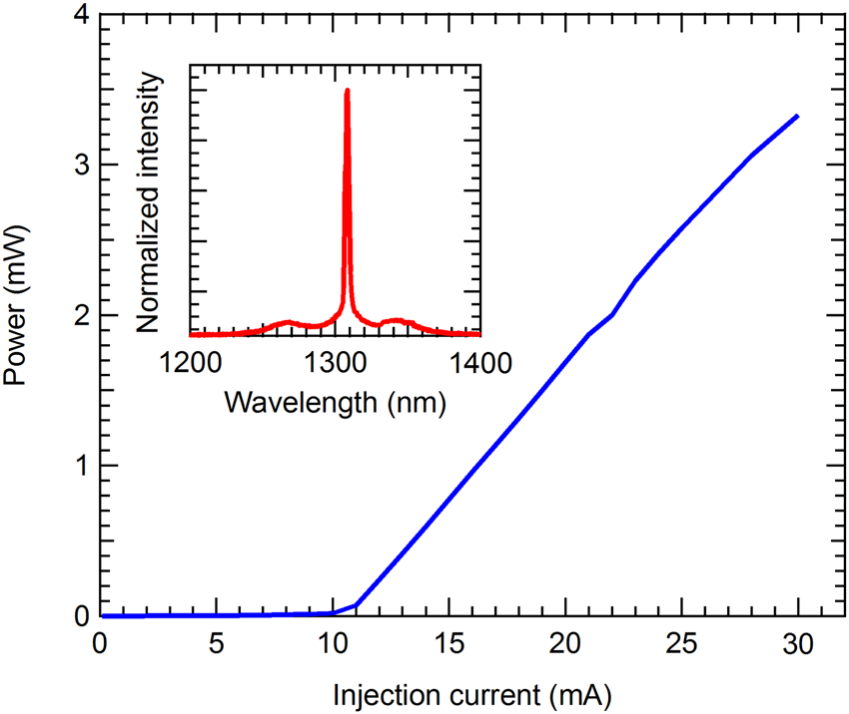
Input-output characteristics of the laser diode used in this study. The inset shows the lasing spectrum of the laser with an input current of 20 mA.

The lasing performance of the laser diode was first investigated with continuous-wave (CW) current injections. Figure 2 shows the plot of the output power against the injection current. It is shown that the laser diode has a threshold current of 11 mA, quantum efficiency of 0.17 W/A, and lasing wavelength of 1310 nm.

**Figure 3:**
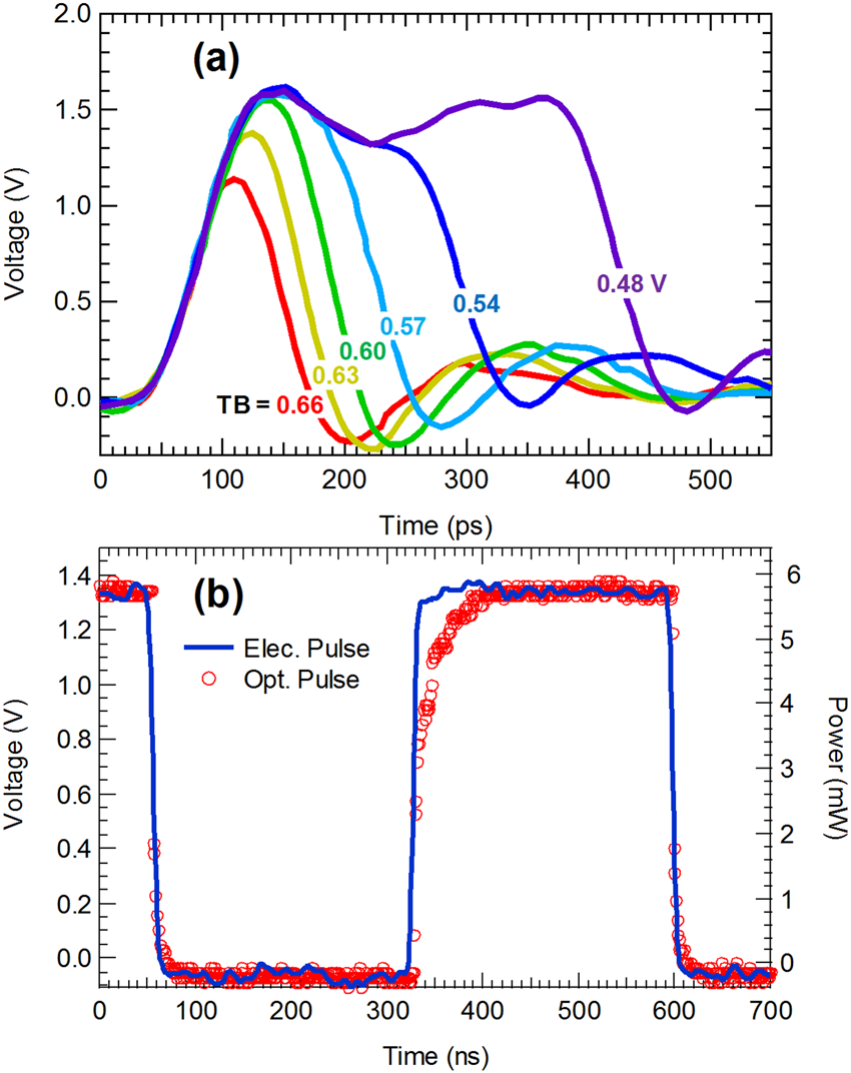
Results of the electrical measurements taken on the integrated CMOS pulser. (**a**) Waveforms of the output pulses with different pulse widths from the CMOS pulser with different voltage of TB from 0.66 to 0.48 V. Each waveform is marked with the corresponding voltage. (**b**) Waveform of the electrical pulse from the CMOS pulser with a duration of 270 ns and the corresponding optical pulse from the laser diode.

The results of the electrical measurements taken on the integrated CMOS pulser are shown in Fig. 3(a) and (b). By tuning the voltage applied to the CMOS (TB), the output pulse width can be tuned continuously up to 270 ns, with a minimum pulse width of 80 ps. The output voltage can be tuned from 0.8 to 1.5 V. Figure 3(b) shows the waveform of an electrical pulse with a duration of 270 ns generated by the CMOS pulser, as well as the corresponding optical output pulse from the laser diode. It can be seen that while the shapes of the electrical and optical pulses differ at the rise edges, the falling edges overlap with each other well, which is a result of the transient gain-switching process (it appears that the optical pulse is “cut” by the falling edge of the electrical pulse). The operating frequency of the system depends on the oscillator used for the initial pulse generation, which is approximately 1.8 MHz. This frequency can therefore be changed by choosing a different oscillator.

**Figure 4:**
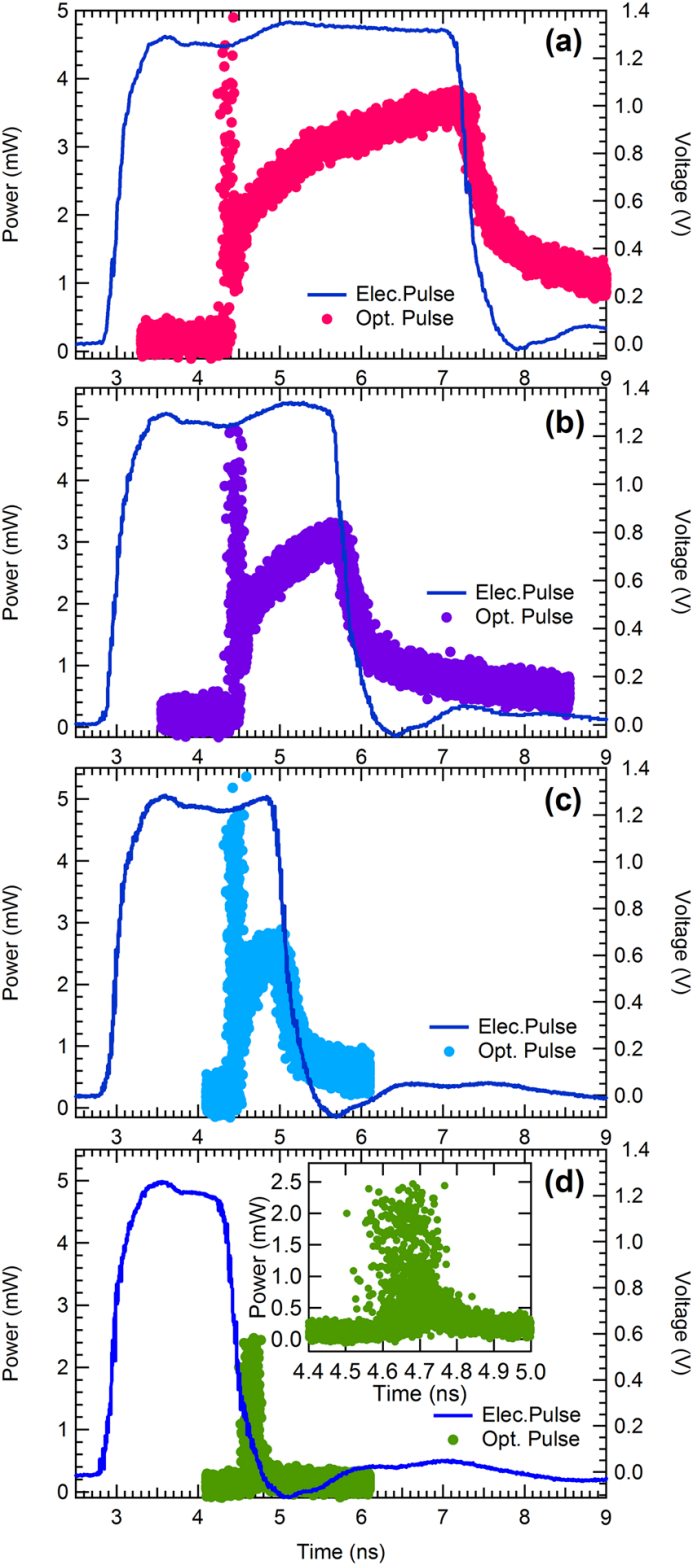
Waveforms of the optical output of the laser diode generated due to the application of electrical pulses with different durations. (**a**) Waveforms of the driving electrical pulse with a duration of 4.3 ns and the corresponding optical pulse with a duration of 3.0 ns. (**b**) Waveforms of the driving electrical pulse with a duration of 2.7 ns and the corresponding optical pulse with a duration of 1.3 ns. (**c**) Waveforms of the driving electrical pulse with a duration of 2.0 ns and the corresponding optical pulse with a duration of 0.7 ns. (**d**) Waveforms of the driving electrical pulse with a duration of 1.7 ns and the corresponding optical pulse with a duration of 0.3 ns. (**e**) Waveforms of the driving electrical pulse with a duration of 1.4 ns and the corresponding optical pulse with a duration of around 100 ps. Inset shows an enlarged figure of the waveform of the optical pulse.

Figure 4 shows the waveforms of optical pulses from the laser diode generated as a result of applying electrical pulses with different durations. The output voltage of the electrical pulses is fixed at 1.4 V. From Fig. 4(a), we can see that the optical pulse consists of two parts, an initial spike followed by a steady state region. This shape is typical of gain-switched optical pulses generated using long electrical pulse excitation^13^. According to our previous research, the delay time of the first spike, which is also called the turn-on time of the laser diode, depends on the gain property of the laser diode^13, 24, 31^. Increasing the gain of the laser diode can thus reduce the turn-on time.

From Fig. 4(a,d), we see that reducing the electrical pulse duration simultaneously reduces (or cuts) the steady state durations of the optical pulses, even though the delay time of the optical pulses remains unchanged. That is to say, the optical pulse width is adjusted by tuning the electrical pulse width. Figure 4(d) shows that this steady state can be significantly reduced, leaving only the initial spike of the optical pulse, which has a minimum pulse-width of approximately 100 ps. The intrinsic minimum duration of the first spike depends on the gain and structural properties of the semiconductor laser diode and can further be reduced by optimizing the parameters of the laser diode. The inset shows an enlarged figure of the waveform of the first spike, indicating that the waveform contains many jitters.

**Figure 5:**
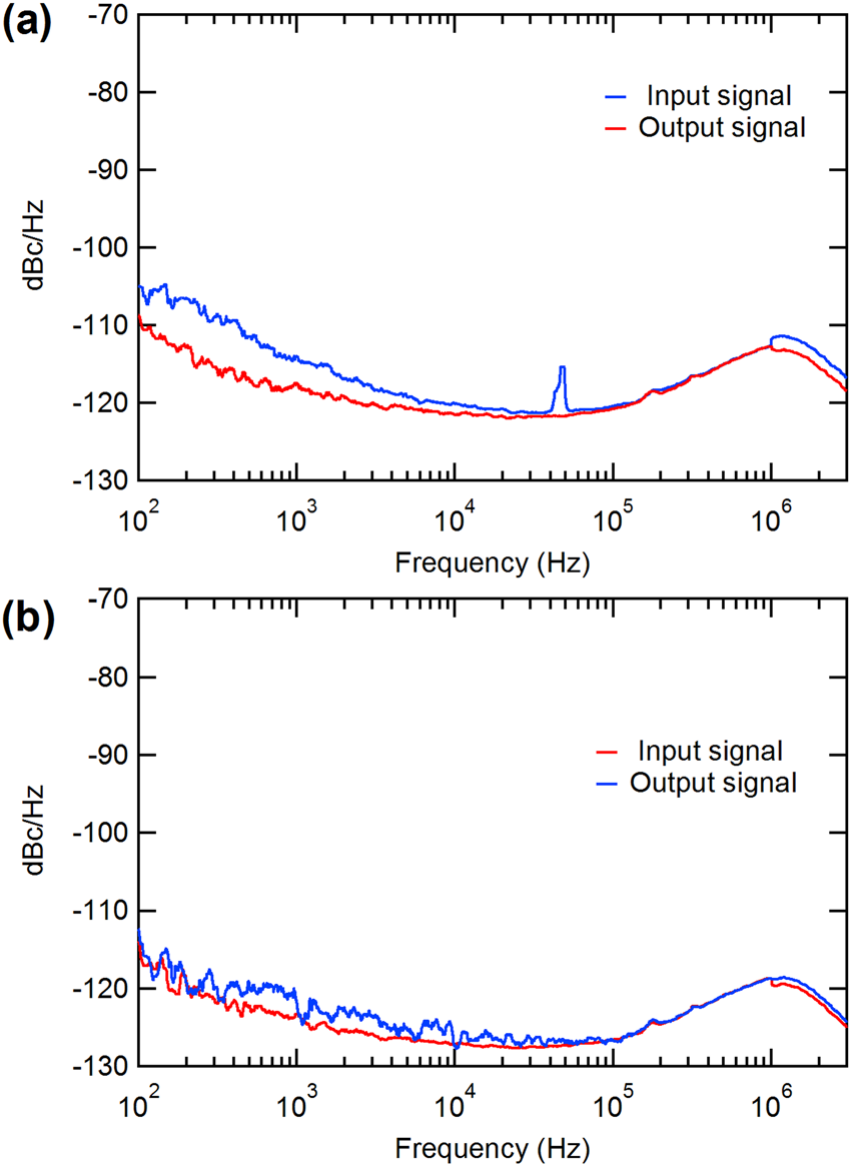
(**a**) Phase noise comparison between the input and output of the pulse generator. (**b**) Phase noise comparison between the input and output of the semiconductor laser diode.

Further experiments on jitter measurements (See Supplementary Information) have been performed. Figure 5(a), shows the phase noises of the input and output signals of the CMOS pulser, respectively. The rms jitters are 34.208 ps and 39.450 ps, respectively, which means the extra jitter contribution by the pulse generator circuit is only 5.242 ps. Meanwhile we also measure the phase noise of the input pulse and the optical outputs of the laser diode, as shown in Fig. 5(b), the rms jitters are 33.421 ps and 35.552 ps, which means the semiconductor laser diodes only contributes a rms jitter of 2.131 ps. Therefore, the jitter is mainly induced by the jitter inherent to the circuits-voltage oscillator. For future implementation, an oscillator of improved quality could be used to reduce the jitter of our laser driving circuit.

**Figure 6:**
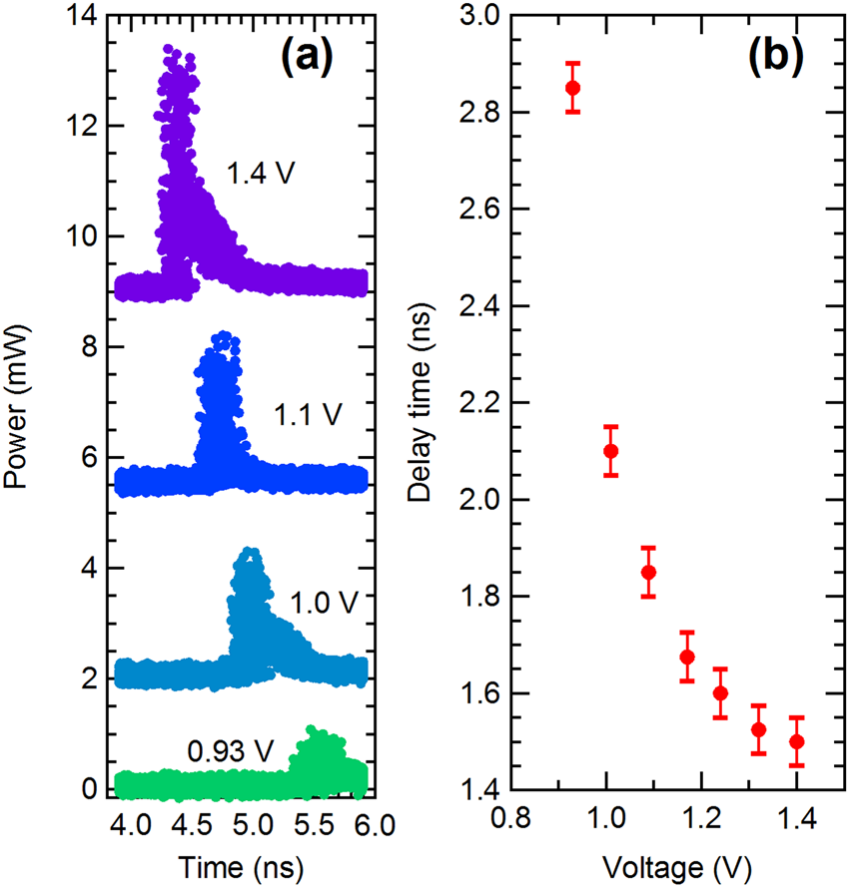
(**a**) Waveforms of the optical pulses from the laser diode generated with different driving voltages ranging from 0.9 to 1.4 V (for easy observation of the pulse shapes, the electrical pulse widths are tuned to cut off the steady state). (**b**) Delay times of the optical pulses from the laser diode generated with different driving voltages ranging from 0.9 to 1.4 V.

The fixed delay time in Fig. 4 results from the fixed driving voltage; thus, changing the driving voltage of the laser results in a change in the delay time. Figure 6(a) shows the waveforms of optical pulses generated with driving voltages ranging from 0.9 to 1.4 V (for easy observation of the pulse shapes, the electrical pulse-widths are tuned to the cut off of the steady state). From Fig. 6(b), we can see that by increasing the driving voltage, the delay time of the optical pulses is reduced, which is also a typical gain-switching property of semiconductor lasers.

**Figure 7:**
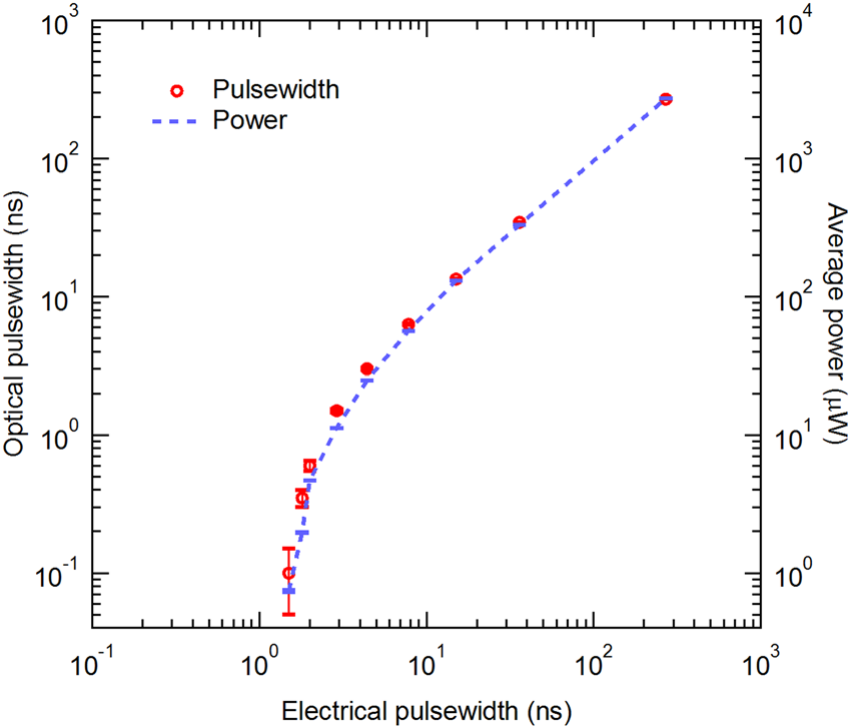
Optical pulse widths and the average optical powers plotted against the electrical pulse width of the whole laser system.

Figure 7 summarizes the tuning ability of the electric pulser driven semiconductor laser system. The tuning range of the electric pulser is 8 ps–270 ns, and consequently, the optical pulse width can be tuned from 100 ps to 270 ns. The peak power of the optical pulse is approximately 4 mW, which is a value suitable for use as seed lasers, for fiber amplifications.

Note that the 100 ps minimum optical pulse width is generated from an electrical pulse width of 1.5 ns, which is much longer than the 80 ps minimum that the electric pulser can achieve. The 100 ps optical pulse width is limited primarily by the gain-switching properties of the semiconductor laser; the long delay time indicates a low-gain semiconductor laser is being used^28, 29^. With proper high-gain and high-speed laser diodes^13, 21^, shorter optical pulses with pulse widths of several picoseconds can be expected using the current electric pulser.

The intrinsic time accuracy for the SMIC CMOS 40-nm process can be as low as 9.4 ps^32^. The internal circuit used can generate a similarly short pulse. However, after significant filtering of package parasitics, high-frequency components are dramatically attenuated. The 80-ps electrical pulse width is therefore limited by the external matching network and package parasitics. Through system integration, or the optimization of PCB and package design, it is possible to generate shorter electrical pulses sufficient for driving semiconductor lasers. Moreover, if we can manage to integrate the pulse generator and the laser diode, the effects of the matching problem and package parasitics can be minimized further.

The current maximum output voltage is approximately 1.5 V and depends on the CMOS gate thickness. If a standard thick-gate CMOS process is adopted, a maximum output voltage of 5 V can be expected; however, the intrinsic pulse width may be broadened slightly to about 10 ps. Furthermore, by adopting the laterally diffused metal oxide semiconductor (LDMOS) technique, an even higher driving voltage (>25 V) may be achieved, which is useful for high power laser diodes.

## Conclusions

We designed and produced a low-cost integrated CMOS pulse generator using a 40-nm microelectronic process technique. We then designed the peripheral circuit and converted the CMOS pulse generator into a pulse-width-tunable electric pulser with a minimum pulse width of 80 ps, as well as a tunable output voltage ranging from 0.9 to 1.5 V and a wide tuning range of up to 270 ns. We used this pulser to drive a 1.3-µm semiconductor laser diode directly and investigated the gain-switching properties of the whole laser system. When tuning the electrical pulse width to approximately 1.5 ns, the semiconductor laser produces optical pulses with a minimum pulse width of 100 ps. The optical pulse widths can be tuned continuously up to 270 ns by adjusting the electrical pulse width. The electric pulser produced can be used to directly drive semiconductor laser diodes with wavelengths in the near-infrared range, which are suitable for power amplification with rare-earth doped fiber amplifiers.

## Electronic supplementary material

Supplementary Information

## References

[1] Sikora A (2010). Structure of diamondlike carbon films deposited by femtosecond and nanosecond pulsed laser ablation. Journal of Applied Physics.

[2] Reenaas TW (2015). Femtosecond and nanosecond pulsed laser deposition of silicon and germanium. Applied Surface Science.

[3] Chen SC (2014). Growth and characterization of Cu(In,Ga)Se2 thin films by nanosecond and femtosecond pulsed laser deposition. Nanoscale research letters.

[4] Sugioka K, Cheng Y (2014). Ultrafast lasers—reliable tools for advanced materials processing. Light: Science & Applications.

[5] Hecht J (2010). A short history of laser development. Applied Optics.

[6] Nee CH (2016). Direct synthesis of nanodiamonds by femtosecond laser irradiation of ethanol. Scientific Reports.

[7] Rehman ZU, Janulewicz KA (2016). Structural transformations in femtosecond laser-processed n-type 4H-SiC. Applied Surface Science.

[8] Kerse C (2016). Ablation-cooled material removal with ultrafast bursts of pulses. Nature.

[9] Oosterbeek RN (2016). Fast femtosecond laser ablation for efficient cutting of sintered alumina substrates. Optics and Lasers in Engineering.

[10] Zhao WQ (2016). Wavelength effect on hole shapes and morphology evolution during ablation by picosecond laser pulses. Optics & Laser Technology.

[11] Auyeung J (1981). Picosecond optical pulse generation at gigahertz rates by direct modulation of a semiconductor laser. Appl. Phys. Lett..

[12] Lanz B, Vainshtein S, Kostamovaara J (2006). High power gain-switched laser diode using a superfast GaAs avalanche transistor for pumping. Applied Physics Letters.

[13] Chen SQ (2012). Sub-5-ps optical pulse generation from a 1.55-µm distributed-feedback laser diode with nanosecond electric pulse excitation and spectral filtering. Optics Express.

[14] Nakazawa M, Suzuki K, Kimura Y (1990). Transform-limited pulse generation in the gigahertz region from a gain-switched distributed-feedback laser diode using spectral windowing. Opt. Lett..

[15] Yamada N, Nakagawa Y (1993). Pulse shaping using spectral filtering in gain-switched quantum well laser diodes. Appl. Phys. Lett..

[16] Wada K (2008). Pulse-shaping of gain-switched pulse from multimode laser diode using fiber Sagnac interferometer. Opt. Express.

[17] Consoli A, Esquivias I (2012). Pulse shortening of gain switched single mode semiconductor lasers using a variable delay interferometer. Opt. Express.

[18] Wang FQ (2016). Stable Gain-Switched Thulium Fiber Laser With 140-nm Tuning Range. IEEE Photon. Technol. Lett..

[19] Zhang BF, He GY, Jiao ZX, Wang B (2016). Efficient theoretical model and numerical simulation for optimization of gain‑switched thulium‑doped fiber lasers. Appl. Phys. B..

[20] Petkovšek R, Agrež V (2014). Single stage Yb-doped fiber laser based on gain switching with short pulse duration. Opt. Express.

[21] Asahara A (2014). Direct generation of 2-ps blue pulses from gain-switched InGaN VCSEL assessed by up-conversion technique. Scientific Reports..

[22] Bimberg D, Ketterer K, Bottcher EH, Scoll E (1986). Gain modulation of unbiased semiconductor lasers: ultrashort light-pulse generation in the 0.8 μm-1.3 μm wavelength range, Int. J. Electronics.

[23] Ryvkin BS, Avrutin EA, Kostamovaara J (2009). Asymmetric-Waveguide Laser Diode for High-Power Optical Pulse Generation by Gain Switching. J. Lightwave Technol..

[24] Vasil’ev, P. Ultrafast diode laser: fundamentals and applications, Artech House Optoelectronics Library, April 30 (1995).

[25] Ransijn H, Salvador G, Daugherty D, Gaynor K (2001). A 10-Gb/s laser/modulator driver IC with a dual-mode actively matched output buffer. IEEE Journal of Solid-State Circuits.

[26] Lange S (2016). Low power InP-based monolithic DFB-Laser IQ Modulator with SiGe Differential Driver for 32-GBd QPSK Modulation. Journal of Lightwave Technology.

[27] Diao S, Zheng Y, Heng CH (2009). A CMOS ultra low-power and highly efficient UWB-IR transmitter for WPAN applications. IEEE Transactions On Circuits and System II.

[28] Nissinen J, Kostamovaara J (2016). A High Repetition Rate CMOS Driver for High-Energy Sub-ns Laser Pulse Generation in SPAD-Based Time-of-Flight Range Finding. IEEE Sensors Journal.

[29] Li DU, Huang LR, Tsai CM (2005). A 3.5Gb/s CMOS Burst-Mode laser driver with automatic power control using single power supply. 2005 IEEE International Symposium on Circuits and Systems.

[30] Siriani DF (2013). Watt-class nanosecond-pulse semiconductor laser with integrated driver. IEEE conference CLEO.

[31] Lau KY (1988). Gain switching of semiconductor injection lasers. Applied Physics Letters.

[32] Liu, X. *et al*. A low power TDC with 0.5 ps resolution for ADPLL in 40 nm CMOS. IEEE 11th International Conference on ASIC (ASICON), Page 1–4 doi:10.1109/ASICON.2015.7517035 (2015).

