# Supplementary material for: Broadband tunable integrated CMOS pulser with 80-ps minimum pulse width for gain-switched semiconductor lasers
Source: Sci Rep. 2017 Jul 31;7:6878. doi: 10.1038/s41598-017-07138-3 (PMC5537295; doi:10.1038/s41598-017-07138-3)
Supplement: Supplementary file 1 — Supplementary Information [file 41598_2017_7138_MOESM1_ESM.pdf]

Supplementary Information

Broadband tunable integrated CMOS pulser with 80-ps minimum pulse width for gain-switched semiconductor lasers

Shaoqiang Chen[1], Shengxi Diao[1], Pengtao Li[1], Takahiro Nakamura[2], Masahiro Yoshita[3], Guoen Weng[1], Xiaobo Hu[1], Yanling Shi[1], Yiqing Liu[1], Hidefumi Akiyama[2].

1. School of Information Science and Technology, East China Normal University, 500 Dongchuan Road, Shanghai 200241, China.
2. Institute for Solid State Physics, The University of Tokyo, 5-1-5 Kashiwanoha, Kashiwa, Chiba 277-8581, Japan.
3. Research Center for Photovoltaics, National Institute of Advanced Industrial Science and Technology, 1-1-1 Umezono, Tsukuba, Ibaraki 305-8560, Japan

1. Pulse Generator

A detailed schematic of the tunable pulse generator is shown in the following figure.

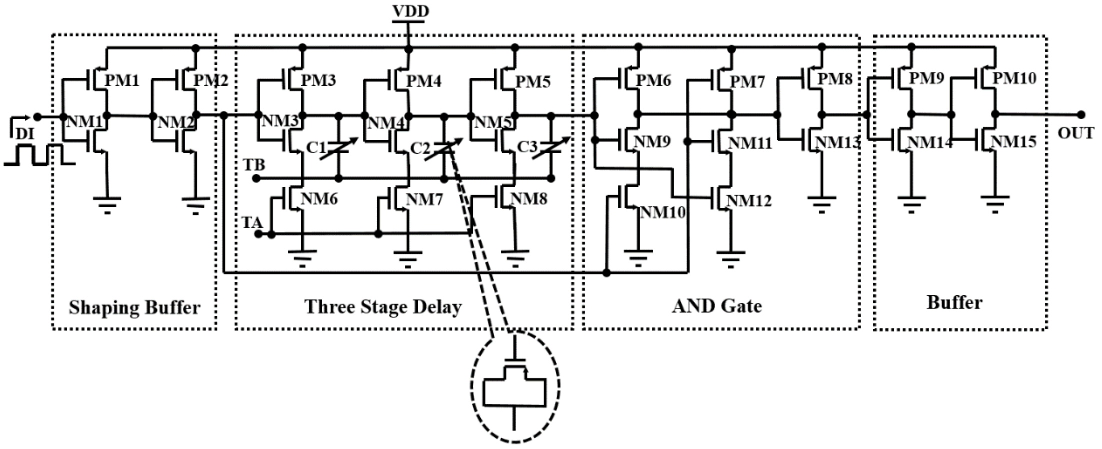

Fig-SI-1. A detailed schematic of the tunable pulse generator.

Here, the MOS transistors PM1-2 and NM1-2 form two inverters, which are used to shape the data input (DI) and make its rise edge and falling edge sharp. As a result, crosstalk and noise along the DI signal flow from external to internal can be removed. After the two inverters, the DI signal is input to a pulse generator, consisting of PM3-8 and NM3-13, where three same delay stages are included. The first stage consists of PM3, NM3, NM6, and C1. The current controlled by an external control voltage (TA) is fed into the gate of the NM6 transistor. The delay can also be tuned by PMOS type varactor C1, the

drain and source terminal of which are connected to external control voltage, TB. The capacitance can be tuned by changing TB. Next, PM6-8 and NM9-13 form an AND gate. With the input signals as DI and the delayed inverted DI, the output is equal to the delay time, which can be tuned from 80 ps to 270 ns by controlling the values of TA and TB. PM9-10 and NM14-15 form the output buffer and send the pulse to the laser through an analog PAD connector (50 μm × 50 μm). The delay can be affected by PVT variation. To rectify this, calibration will be performed using a digitally controlled binary-weighted current source or capacitance to control the equivalent resistance, *R* or capacitance, *C* in upcoming prototypes. Detailed sizes of the transistors are listed in the following table.

Table-SI-1. Detailed sizes of the transistors in the circuit

	W(μm)	L(μm)		W(μm)	L(μm)		W(μm)	L(μm)
PM1	10	0.04	NM1	4	0.04	NM14	54	0.04
PM2	30	0.04	NM2	12	0.04	PM10	300	0.04
PM3-5	40	0.04	NM3-5	16	0.04	NM15	120	0.04
PM6-7	60	0.04	NM6-8	60	0.04	C1-3	2	2
PM8	90	0.04	NM9-12	24	0.04			
PM9	135	0.04	NM13	36	0.04			

2. Jitter measurements

During the jitter measurement, the CMOS pulse generator was triggered with a 20-MHz square wave generated from an arbitrary waveform generator (ROHDE&SCHWARZ HMF2550 50 MHz). The jitter and phase noise of the CMOS pulse generator were measured with a phase noise analyzer (ROHDE&SCHWARZ FSWP-PHASE NOISE ANALYZER 1 MHz - 50 GHz). As shown in the following figure (Fig-SI-2), the electrical pulses follow the electrical trigger well, except below 10 kHz, which should be the CMOS circuits' flicker noise contribution. The integrated RMS jitter of the trigger signal is 34.208 ps (trace 2 in Fig-SI-2) and the RMS jitter of the electrical pulses is 39.45 ps (trace 1 in Fig-SI-2). The incremental of 5.24-ps jitter is not big, the electrical output jitter follows the trigger signal's jitter well. Therefore, the jitters of the electrical pulse in the present system mainly come from the crystal oscillator. In order to improve the performance of the CMOS pulse generator, it is very useful to use high precise crystal oscillators with low jitter as triggers.

The optical jitter is also related to the electrical pulse jitter. It is verified through the experimental results as shown in Fig-SI-3. The input signal of the laser diode for the jitter

measurement is a 10-MHz electrical square wave generated from the arbitrary waveform generator (R&S HMF2550 50MHz). The optical output pulse from the laser diode was converted to electrical signal through a photodetector (PD1014, Newport). The jitter of the input electrical square wave and the optical output of the laser diode were then measured to be 33.42 ps (Trace 1) and 35.55 ps (Trace 2), respectively, through the phase noise analyzer (R&S FSWP). Therefore, the optical jitter follows the electrical jitter quite well. In order to improve the performance of the system, a high precise crystal oscillator should be applied to achieve good jitter performance at the original trigger input.

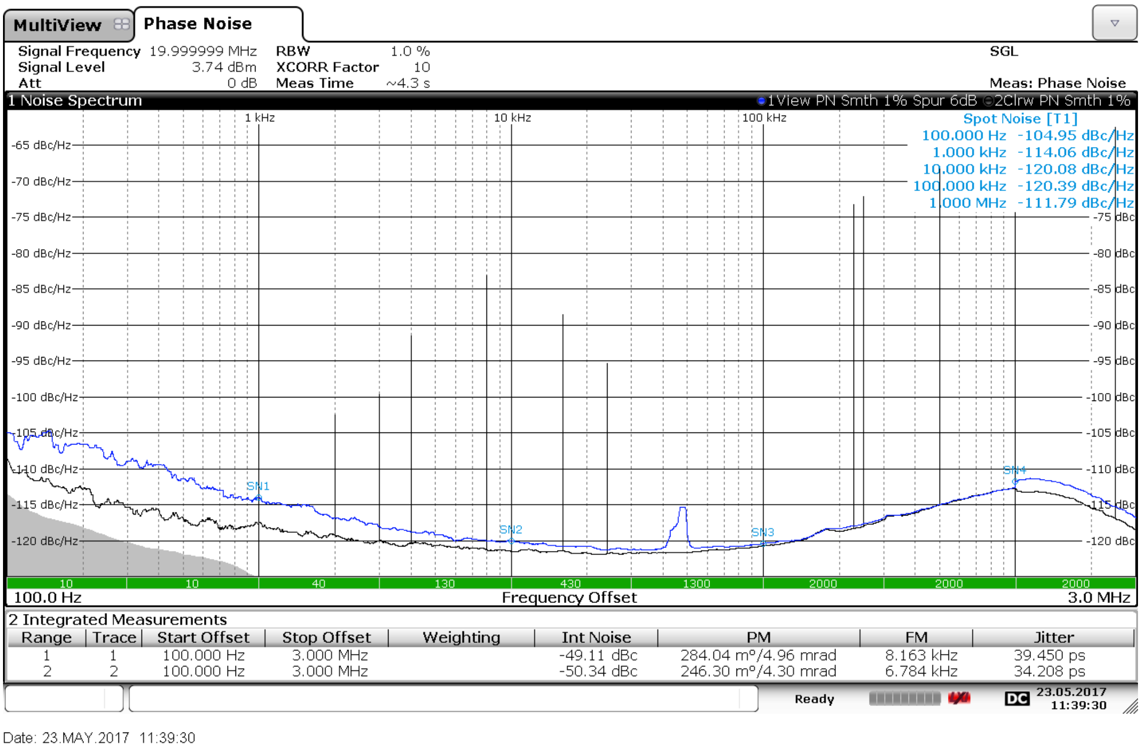

Fig-SI-2. Screen image of the phase noise analyzer (R&S FSWP) during the jitter measurement of CMOS pulse generator (Trace 2) and the trigger signal (Trace 1) from the arbitrary waveform generator (R&S HMF2550).

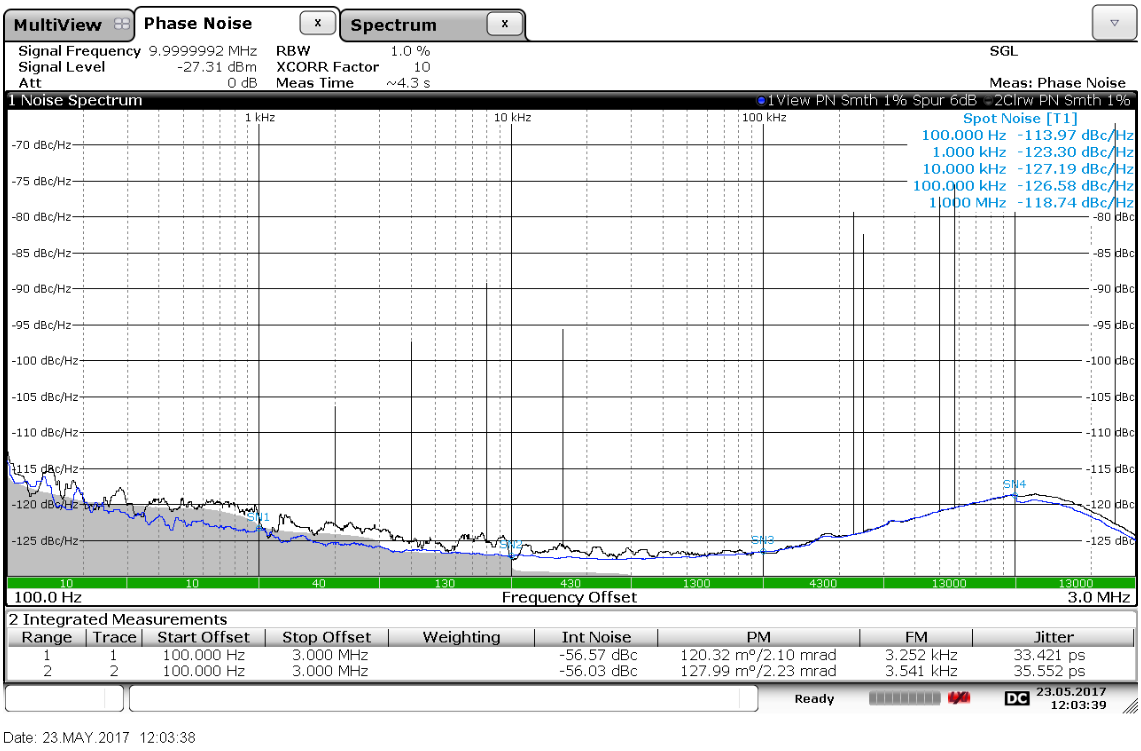

Fig-SI-3. Screen image of the phase noise analyzer (R&S FSWP) during the jitter measurement of optical pulses from the laser diode (Trace 2) and the trigger signal (Trace 1) from the arbitrary waveform generator (R&S HMF2550).
